# Effect of quorum sensing signals produced by seaweed-associated bacteria on carpospore liberation from *Gracilaria dura*

**DOI:** 10.3389/fpls.2015.00117

**Published:** 2015-03-04

**Authors:** Ravindra Pal Singh, Ravi S. Baghel, C. R. K. Reddy, Bhavanath Jha

**Affiliations:** ^1^Seaweed Biology and Cultivation Group, Division of Marine Biotechnology and Ecology, CSIR-Central Salt and Marine Chemicals Research InstituteBhavnagar, India; ^2^Academy of Scientific and Innovative Research (AcSIR)New Delhi, India

**Keywords:** quorum sensing, carpospores liberation, *Gracilaria dura*, *Vibrio*, *Ulva* spp.

## Abstract

Epiphytic and endophytic bacteria associated with green macroalgae *Ulva* (*U. fasciata* and *U. lactuca*) and red macroalgae *Gracilaria* (*G. corticata* and *G. dura*) have been identified from three different seasons to evaluate the effect of quorum sensing (QS) molecules on carpospores liberation from *Gracilaria dura*. The bacterial isolates belonging to the orders *Bacillales, Pseudomonadales, Alteromonadales*, and *Vibrionales* were present in all seasons, whereas *Actinomycetales* and *Enterobacteriales* were confined to pre-monsoon and post-monsoon seasons, respectively. Among all the Gram-negative bacteria, seven isolates were found to produce different types of *N*-acyl homoserine lactones (AHLs). Interestingly, *Shewanella algae* produced five types of AHL: C_4_-HSL, HC_4_-HSL, C_6_-HSL, 3-*oxo*-C_6_-HSL, and 3-*oxo*-C_12_-HSL. Subsequently, the AHLs producing bacterial isolates were screened for carpospore liberation from *G. dura* and these isolates were found to positively induce carpospore liberation over the control. Also, observed that carpospore liberation increased significantly in C_4_- and C_6_-HSL treated cystocarps. Sodium dodecyl sulfate and native polyacrylamide gel electrophoresis of the total protein of the C_4_- and C_6_-HSL treated cystocarps showed two specific peptide bands of different molecular weights (50 kDa and 60 kDa) as compared to the control, confirming their indirect effect on carpospore liberation.

## Introduction

Extracellular substances released from macroalgal communities serve as feed for diverse microorganisms in coastal ecosystems (Armstrong et al., 2001; Lane and Kubanek, 2008). Microbial communities living on macroalgal surfaces are highly diverse, complex and dynamic and they consist of a consortium of microorganisms (Holmström et al., 2002). However, bacteria are the most ubiquitous, occurring on the external surfaces and in the internal tissues of the algae (Hollants et al., 2011). Macroalgal bacterial communities have been found to play an important role in the growth, development, morphogenesis, and reproduction of the green macroalga *Ulva* (Patel et al., 2003; Matsuo et al., 2005; Tait et al., 2005; Joint et al., 2007; Singh and Reddy, 2014). The green macroalga *Ulva* forms an aberrant morphology instead of the typical foliose thallus morphology when cultured axenically (Provasoli and Pintner, 1980). This aberrant morphology is successfully reversed to the foliose thallus morphology following the inoculation of appropriate morphogenesis-inducing bacteria to the culture medium (Nakanishi et al., 1996; Singh et al., 2011a). Additionally, macroalgae-associated bacterial isolates of epi- and endophytic origin have been reported to produce indole-3-acetic acid (IAA) that regulates morphogenesis pattern and growth in *Ulva* spp. (Maruyama et al., 1988) and *Gracilaria dura* (Singh et al., 2011b). Several studies have revealed that bacterial groups belonging to *Proteobacteria, Firmicutes*, and *Actinobacteria* are commonly associated with the *Ulva* and *Gracilaria* species (Patel et al., 2003; Tait et al., 2005; Burke et al., 2011; Lachnit et al., 2011). Furthermore, it has been found that consistent detection of these bacterial communities may have a more important functional role in the life processes of the *Ulva* and *Gracilaria* species. Therefore, the characterization of epi- and endophytic bacterial communities and further evaluation of the effect, they have on their hosts is of paramount importance in the ecophysiology of macroalgae.

It has also been established that macroalgae-associated bacterial isolates produce quorum sensing (QS) signal molecules, such as *N*-acyl homoserine lactone (AHLs), thereby facilitating the settlement of zoospores in *Ulva* spp. (Joint et al., 2002, 2007; Williams, 2007). Joint et al. (2002) established that AHLs producing a *Vibrio anguillarum* biofilm positively enhanced the settlement of zoospores of the *Enteromorpha* species. Tait et al. (2005) studied the stability and diffusion rate of AHLs produced from *V. anguillarum* biofilm and found that AHLs with longer *N*-acyl side-chains tended to result in increased zoospore settlement of *Ulva*. Further investigation of zoospore settlement revealed that the orientation of zoospore does not change during settlement (Wheeler et al., 2006). The mechanism underlining this phenomenon has not yet been reported; however, it has been assumed that AHLs influence Ca^2+^ influx in zoospore which preferentially induces the settlement through chemokinesis (Wheeler et al., 2006). Interestingly, the effect of AHLs was also observed in the red alga *Acrochaetium* sp. (Weinberger et al., 2007). That study found that C_4_-HSL has the ability to induce the carpospores' liberation from *Acrochaetium* sp. (Weinberger et al., 2007). However, the study did not identify AHLs producing host-associated bacteria. Thus, there is limited knowledge about the significant role of cross-kingdom QS signaling between associated bacterial communities and carpospore liberation from red macroalgae.

Cross-kingdom QS signaling between plant roots and their rhizospheric bacteria has also been demonstrated (Hartmann et al., 2014). For example, AHLs produced from symbiotic bacteria elicited developmental changes in the root system (Ortíz-Castro et al., 2008) and root stimulatory effect in *Arabidopsis* (Jin et al., 2012; Liu et al., 2012). Götz et al. (2007) has found that C_6_-, C_8_- and C_10_-HSL altered root and shoot growth in *Hordeum vulgare*. Recently, Veliz-Vallejos et al. (2014) demonstrated that 3-oxo-C_14_-HSL from *Sinorhizobium meliloti* increased nodule numbers in *Medicago truncatula*. Some studies have also been carried out to understand the role of AHLs in plant defense (Hartmann et al., 2004; Schuhegger et al., 2006). *Serratia liquefaciens* MG1 produces C_4_- and C_6_-HSL and is found to induce specific systemic resistance proteins after the roots were inoculated with the bacterium (Hartmann et al., 2004). *S. meliloti* specifically enhances the resistance of *A. thaliana* toward the pathogens *Pseudomonas syringae* and *Golovinomyces orontii* and the resistance of *H. vulgare* and *Blumera graminis* (Schikora et al., 2011; Schenk et al., 2012; Zarkani et al., 2013).

*Ulva* and *Gracilaria* are the most common types of macroalgae and they grow abundantly in intertidal regions of coastal habitats worldwide. The present study has investigated the epi- and endophytic bacteria associated with the *Ulva* and *Gracilaria* species from two different locations and three different seasons in order to identify the bacterial isolates that play a significant role in carpospore liberation. Subsequently, all the isolated bacteria were preliminary screened for their ability to produce AHLs using ESI-MS and the positive isolates were further analyzed using LC-ESI-MS/MS-collision-induced dissociation (CID) to qualitatively analyse the type of AHL. The AHLs producing bacteria were then screened for their potential to liberate carpospores from the red macroalga *G*. *dura*. All the bacterial isolates obtained in this study were identified by 16S rRNA gene sequencing.

## Materials and methods

### Chemicals

QS signaling molecules, such as *N*-acyl-homoserine-lactone, *N*-butanoyl- (C_4_-HSL), *N*-3-hydroxybutanoyl- (HC_4_-HSL), *N-hexanoyl*- (C_6_-HSL), *N*-heptanoyl- (C_7_-HSL), *N*-octanoyl- (C_8_-HSL), *N*-decanoyl- (C_10_-HSL), *N*-dodecanoyl- (C_12_-HSL), *N*-3-oxo-hexanoyl- (3-*oxo*-C_6_-HSL), *N*-3-oxo-octanoyl-(3-*oxo*-C_8_-HSL), and *N*-3-oxo-dodecanoyl-(3-*oxo*-C_12_-HSL) homoserine lactone, were procured from Sigma Aldrich (Buchs, Switzerland). Analytical grade acetonitrile and formaldehyde were purchased from Sisco Research Pvt. Lit. (India). Working concentrations of the AHLs were prepared by dissolving them in acetonitrile (CH_3_CN) at a concentration of 1 mg/ml and then storing them at −20°C.

### Collection of samples and isolation of epiphytic and endophytic bacterial isolates

**Figure 1:**
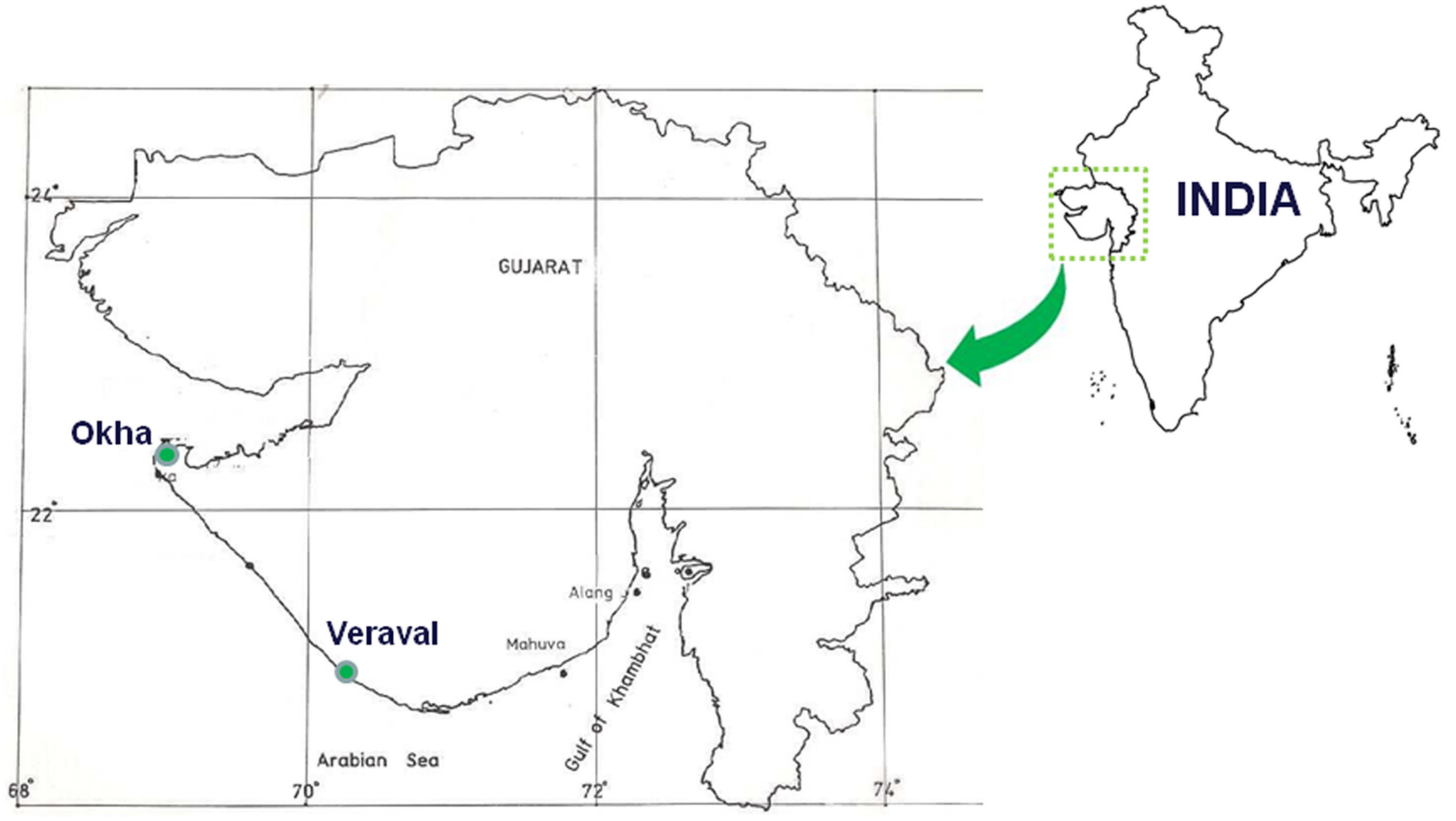
**Map of Gujarat showing macroalgal sampling locations**. The land mass showing collection spots is flanked by Gulf of Kutch on northern part (Okha) and Gulf of Khambhat (Veraval) on southern part of Gujarat (Northern west coast), India.

*Ulva fasciata, U*. *lactuca, Gracilaria dura* and *G*. *corticata* were collected from the Veraval coast of India (N 20° 54.87′, E 70° 20.83′). Two samples*, U. fasciata* and *G. dura*, were also collected from Okha Port sites in India (22° 28′ 22″ N and 69° 05′ 03″ E). Neither *U. lactuca* nor *G*. *corticata* were found at the Okha Port locations. Samples were collected during the low tide periods in three different seasons in 2011. Both sites are located 250 km from each other (Figure 1). The pH, temperature and salinity of the seawater were measured during each collection time (Supplementary Table 1). Three individual plantlets of each species were collected from different three intertidal tide pools spread at least <25 m away from each other. The collection of the macroalgal samples and the isolation of the associated bacteria were carried out using the same procedure as previously described by Singh et al. (2011a,b). In brief, the macroalgal fronds were gently cleaned in autoclaved seawater (ASW) and then a small portion of the frond was placed into different bacterial media [Zobell marine (ZM) agar 2216, Simmons citrate (SC), thiosulfate citrate bile salts sucrose (TCBS), xylose, lysine, deoxycholate (XLD) agar and pseudomonas agar] and incubated at 25 ± 1°C for 2–15 days to isolate the epiphytic bacteria. To isolate the endophytic bacteria, the fronds of *Ulva* and *Gracilaria* spp. were surface-sterilized with different concentrations of surfactant (liquid detergent, 1 and 2% in seawater for 10 min for *Ulva* and *Gracilaria* respectively), oxidizing agents (betadine, 1 and 2% in seawater for 2 min for *Ulva* and *Gracilaria* respectively) and an antibiotic mixture (penicillin-G- 1 g, gentamycin- 1 g, streptomycin sulfate- 2 g, kanamycin- 1 g, neomycin- 200 mg, nystatin- 50 mg) of 1% in seawater for 24 h for *Ulva* and *Gracilaria*, and then incubated at 25 ± 1°C (Singh, 2013). To test the efficacy of the treatment's ability to obtain the surface-sterilized material, the surface-sterilized macroalgal plantlets (four replicates for each sample) were individually placed on different bacterial media, as mentioned above. The surface-sterilized macroalgal plantlets were crushed to fine tissues using a mortar and pestle. Thereafter, up to 10 ml of fine slurry was made using ASW and 100 μl aliquots of it were spread onto the different bacterial media as mentioned above. Different colonies were picked off and re-streaked on the respective media in order to obtain a pure colony. The pure bacterial colonies were maintained at 4 ± 1°C in slants as stock for further experimentation.

### 16S rRNA gene amplification and sequencing

The genomic DNA of different bacteria was extracted using the cetyltrimethylammonium bromide buffer [CTAB 2%, NaCl 1.4 mM, EDTA 50 mM, Tris 100 mM, PVP 20%] method (Chen and Kuo, 1993). Purification of genomic DNA was confirmed with 0.8% agarose gel electrophoresis. The universal 16S rRNA primers 27F and 1492R were used for PCR amplification and sequencing (Lane, 1991). The reaction mixture and PCR conditions were the same as previously described (Singh et al., 2011a). In brief, the PCR reaction mixture contained 2.5 μl 10 × PCR buffer with MgCl_2_, 25 mM of each deoxynucleoside triphosphate (dATP, dCTP, dGTP, dTTP), 100 ng of each of the forward and reverse primers, 1 unit of Taq DNA polymerase and 10 ng of template DNA. The PCR protocol included a 5-min initial denaturation at 95°C, followed by 30 cycles at 94°C for 40 s, 55°C for 40 s, and 72°C for 2 min, with a final cycle of 10 min at 72°C. The amplified products were analyzed on 1.2% (w/v) agarose gels stained with ethidium bromide and the bands were visualized under UV light. The PCR products were purified using a QIAquick PCR purification kit (QIAGEN, no. 28104). The sequences were manually trimmed and their sequence homology was checked against other sequences available at the NCBI GenBank. The sequence alignment of 16S rRNA was carried out by ClustalW2 software (http://www.ebi.ac.uk/Tools/msa/clustalw2/) and the aligned sequences were clustered into operational taxonomic units (OTUs) at 0.03 cut off values using sequence homology. Finally, the aligned 16S rRNA bacterial sequences were used to construct the phylogenetic trees with the neighbor joining method using the MEGA-5 software (Tamura et al., 2011). The bootstrap test was performed with 1000 replicates in the phylogenetic trees. The sequences were taxonomically classified using the Ribosome Database Project (RDP) using Naive Bayesian rRNA classifier version 2.4 with an 80% confidence threshold (Wang et al., 2007).

### AHL production, separation and identification

For the AHL detection, a pure single colony of each Gram-negative bacteria was separately inoculated in a conical flask containing 150 ml Zobell Marine Broth and incubated at 25 ± 1°C overnight on an orbital shaker at 150 rpm. On the following day, an aliquot of 50 ml culture medium was centrifuged at 4000 rpm for 15 min, then the supernatant was collected and the pH was adjusted to 2.5 using 1 N HCl to prevent hydrolysis of the AHLs. The supernatants were mixed with an equal volume of ethyl acetate to extract the AHLs. This step was repeated again to recover the AHLs from the supernatant. The upper organic layer was separated and washed with an equal volume of Milli-Q water. Thereafter, the upper organic layer was again collected and concentrated under nitrogen gas (Shaw et al., 1997). The residues were finally dissolved in 1 ml of 25% methanol containing 0.1% acetic acid and used for analysing the samples with liquid chromatography electrospray ionization mass/mass spectrometry (LC-ESI-MS/MS) and ESI-MS.

The preliminary screening of the samples was first accomplished with ESI-MS, which was then followed by LC-ESI-MS/MS-CID. The characteristics of the ion products were proposed on the basis of low-resolution MS/MS spectra (Morin et al., 2003). The spectra of LC-ESI-MS/MS were recorded from 0 m/z to 300 m/z to obtain definite identification of these ion products for their accurate mass values. The theoretical masses of the most likely AHLs in the protonated form were calculated and compared with standards. ESI-MS and LC-ESI-MS/MS-CID were performed using a Waters® Micromass® Q-Tof micro™ mass spectrometer connected with a Waters alliance HPLC and equipped with an electrospray ionization source. For ESI-MS, the samples were directly injected into the mass spectroscopy and the flow rate was 20 μl/min. Throughout the analysis, the capillary voltage, sample cone and extraction cone were maintained at 2.5 KV, 25 V, and 1.5 V, respectively. For LC-ESI-MS/MS, 20 μl sample residues were injected onto a reverse phase C18 column (Phenomenex, 150 mm × 4.6 mm) and run with a different solvent gradient (Supplementary Table 2). Argon gas was used as the collision source.

### Effect of the bacterial supernatant and the AHL standard on carpospore liberation from *G. dura*

The healthy and mature cystocarpic thalli of *G. dura* were collected from the intertidal region of the Veraval coast on the western side of India and brought to the laboratory in cold seawater (Figure 1). The thallus-bearing cystocarpic structure was cleaned and surface-sterilized following the protocol aforementioned. Thereafter, the surface-sterilized thalli were maintained in conical flasks with sterilized MP 1 medium at 25 ± 1°C under daylight white fluorescent lamps at 15 μ mol photon m^−2^ s^−1^ irradiance with 12:12 h light: dark photoperiod. The plantlets bearing five cystocarps were placed into Petri dishes containing 15 ml of 30% ASW and they were allowed to liberate the carpospores naturally for 7 days. After the carpospores were naturally liberated, the cystocarp-bearing plantlets were treated with different standards of AHLs (C_4_-, C_6_-, C_8_-, C_10_- and 3-oxo-C_12_-HSL) at a concentration of 10 μM each. The different concentrations (2, 4, 6, 8, and 10 μM) of the effective C_4_- and C_6_-HSLstandards were also used to determine the dose dependency of the AHLs for carpospore liberation.

A culture filtrate of different AHLs producing Gram-negative bacteria and *Bacillus flexus* were also used to examine the effect on carpospore liberation. Culture supernatant was collected from an overnight cell culture (Zobell Marine Broth) after centrifugation at 10000 rpm for 2 min. Subsequently, the supernatant was filter sterilized (syringe filters, 0.22 μm, Millipore) and used for the experiments. The experimental set up and the culture condition were maintained in the same way as mentioned in above paragraph, but sterilized culture filtrates were added instead of standard AHLs. Petri dishes containing fronds but no supplementation of AHLs and without added bacterial culture filtrates were treated as the control. We used also used acetonitrile as negative control. All the experiments were carried out in triplicate. The plantlets were transferred to new Petri dishes every 24 h and the liberated carpospores were counted manually using an inverted microscope. The data were represented in average release per mm^2^. One Way ANOVA and Dunnett's *post-hoc* analysis were used to analyse the effect of bacterial culture filtrates and AHLs on carpospore liberation; significant differences were determined at *p* > 0.01. Bonferroni correction was also applied at *p* < 0.001 and *p* > 0.05. Letter designation format was carried out with Tukey's HSD (honestly significant difference) using JMP software, which means sharing the same letters were not different at *p* < 0.05.

### Electrophoresis of protein profile of the AHL-treated cystocarps and the cystocarp-bearing plantlets

To evaluate the effect of the C_4_-, C_6_-, C_8_-, C_10_-, and 3-oxo-C_12_-HSL on the protein profile of the surface-sterilized cystocarps and the cystocarpic plantlets of *G. dura*, the surface-sterilized cystocarps and cystocarpic plantlets were treated with different concentrations of AHLs in conical flasks and kept at 25 ± 1°C for 48 h. Thereafter, the total protein of the control and the different AHL-treated cystocarps and cystocarpic plantlets were extracted by homogenizing 0.2 g fresh weight in 1 ml of the extraction buffer containing 0.5 M Tris–HCl (pH 8.0), 0.7 M sucrose, 50 mM ethylenediaminetetraacetic acid (EDTA), 0.1 M KCl, 2% (v/v) β-mercaptoethanol, and 2 mM phenylmethylsulfonyl fluoride under cool conditions. The homogenates were centrifuged at 12,000 rpm for 20 min at 4°C. The total proteins extracted from the different sources were stored at −20°C for use in further experiments. The protein concentration was determined by Folin's phenol method (Lowry et al., 1951).

The extracted proteins were analyzed with 10% sodium dodecyl sulfate polyacrylamide gel electrophoresis (SDS-PAGE) according to Laemmli (1970). The 20 μg of the total protein extracted from the different AHL-treated cystocarps and cystocarpic plantlets were loaded into gels along with the control. Next, 10% Native-PAGE was used to confirm the results of SDS- PAGE. The protein bands were developed by the silver staining method.

### Accession numbers

The bacterial sequences reported in the present study were submitted to GenBank with the following accession numbers: JQ665283-JQ665389, JN996469, JQ408391, JQ408396, JQ613503- JQ613504, and JQ613506, for the 16S rRNA gene sequences.

## Results

### Taxonomic classification and phylogenetic analysis of the bacteria

**Figure 2:**
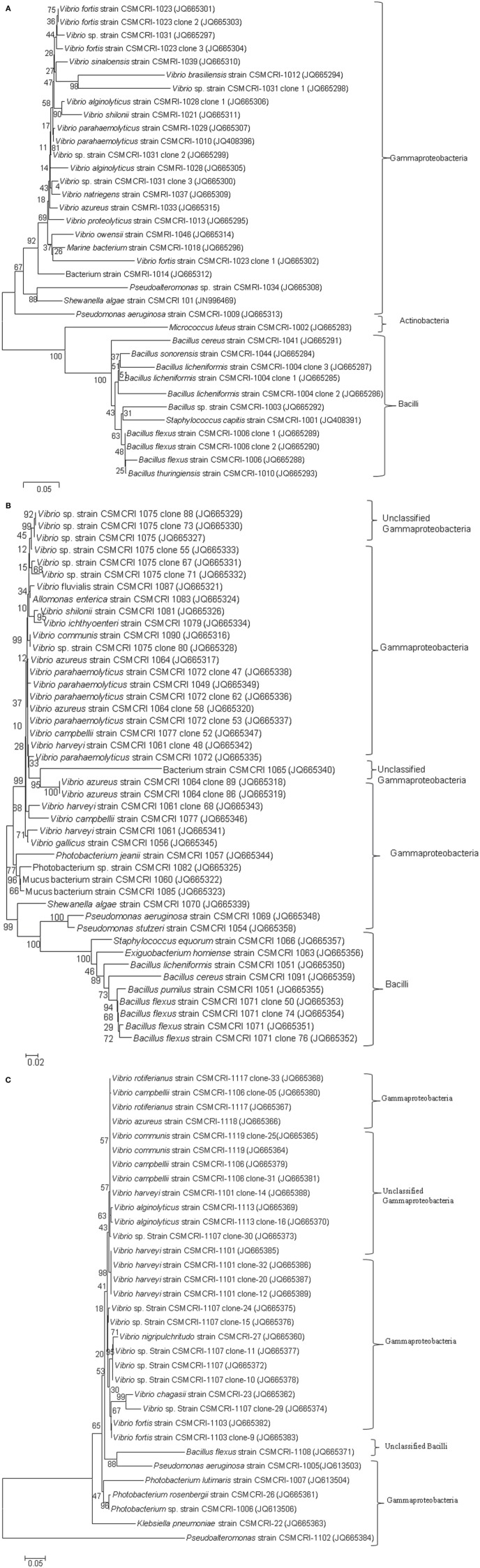
**Phylogenetic relationships of bacterial communities isolated from *Ulva* and *Gracilaria* species during pre-monsoon (2A), monsoon (2B), and post-monsoon (2C) seasons in 2011**. Neighbor-Joining method used for 16S rRNA analysis (Saitou and Nei, 1987). Bootstrap test was performed with 1000 replicates in the phylogenetic trees (Felsenstein, 1985). The tree is drawn to scale, with branch lengths in the same units as those of the evolutionary distances used to infer phylogenetic trees. The evolutionary distances were computed using the Kimura 2-parameter method (Kimura, 1980) and are in the units of the number of base substitutions per site. The rate variation among sites was modeled with a gamma distribution (shape parameter = 5 for pre-monsoon post-monsoon and 2 for monsoon season). Phylogenetic analyses were conducted in MEGA5 (Tamura et al., 2011).

**Figure 3:**
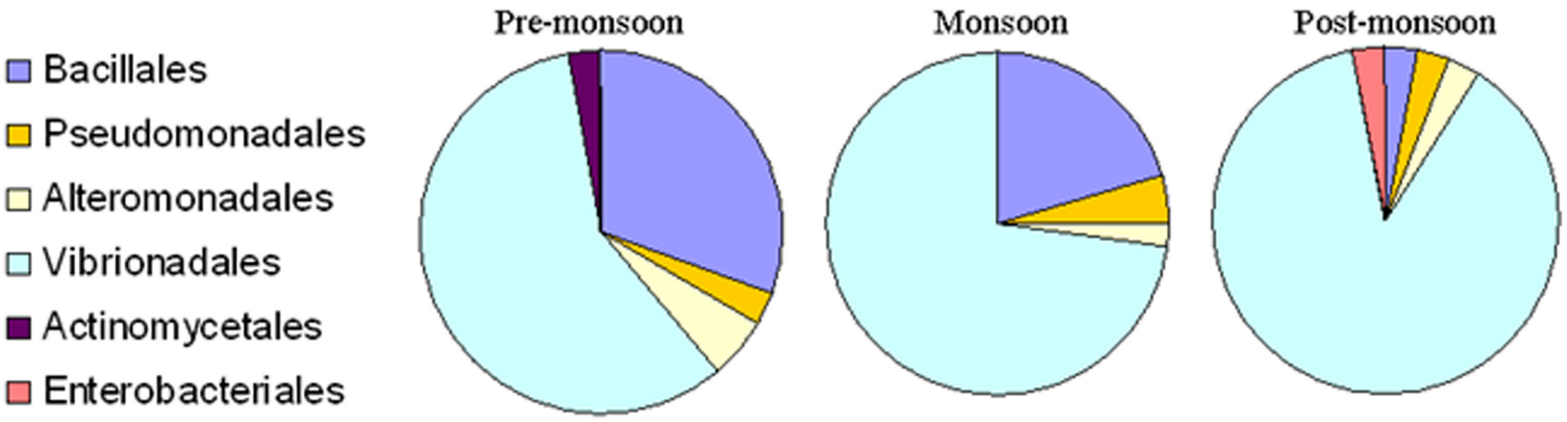
**Percentage composition of different bacterial communities which were isolated from *Ulva fasciata, U*. *lactuca, Gracilaria dura*, and *G*. *corticata***. Samples were collected during low tide periods in three different seasons in 2011.

The present study did not include any short, chimeric or repeated nucleotide sequences. Thus, all the bacterial nucleotide sequences were used to construct the phylogenetic trees. A greater proportion of sequences belonged to the *Gammaproteobacteria*, particularly *Vibrionales*, followed by *Bacillales*, during the pre-monsoon and monsoon seasons. The 87.87% proportion of bacteria collected during the post-monsoon season only belonged to the *Vibrionaceae* family (Figures 2, 3). The phylogenetic trees of the 16S rRNA sequences revealed the proper affiliation of the bacteria that were not properly assigned by the RDP analysis (Figure 2A).

A total of 77 OTUs (≥97% sequence identity) were obtained from all the bacterial nucleotide sequences. The OTUs for the pre-monsoon, monsoon and post-monsoon seasons were 20, 32, and 27, respectively. All of the OTUs represent six orders from three bacterial phyla: *Bacillales, Pseudomonadales, Alteromonadales, Actinomycetales, Enterobacteriales*, and *Vibrionales*. Among these, the bacterial species belonging to *Actinomycetales* (*Micrococcus luteus*) and *Enterobacteriales* (*Klebsiella pneumoniae*) were only found during the pre-monsoon and post-monsoon seasons, respectively (Figure 3).

### Epiphytic and endophytic bacterial isolation

**Figure 4:**
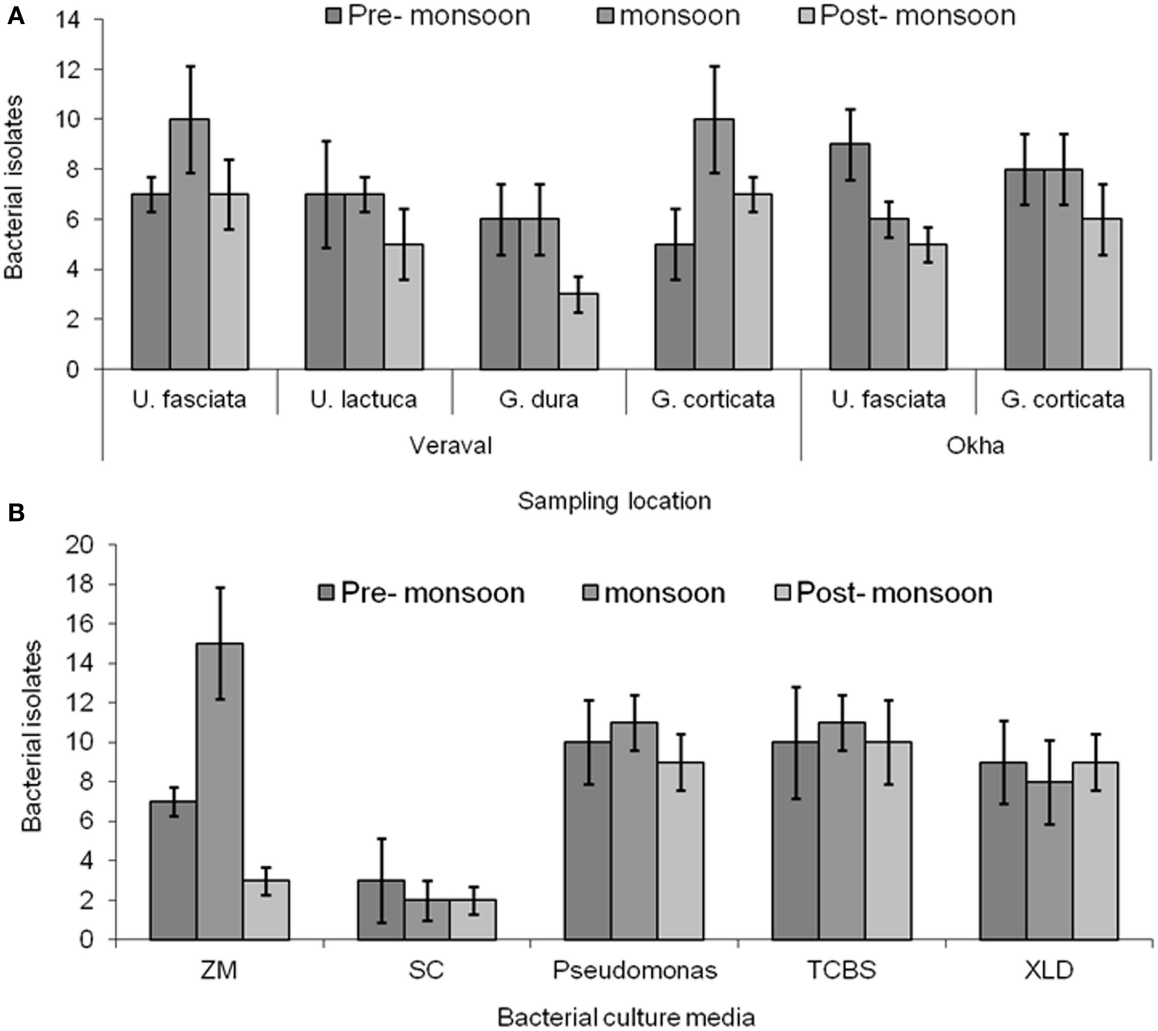
**Bacterial isolation**. **(A)** Enumeration of bacteria from different macroalgal samples such as *Ulva fasciata, U*. *lactuca, Gracilaria dura* and *G*. *corticata*. **(B)** Small plantlets of macroalgae were placed on the different culture media for isolating bacteria from them. Bars indicate, deviation of three independent replicates.

A number of epiphytic bacteria were isolated from seaweeds collected from different locations and during different seasons (Figures 4A,B, Supplementary Table 3). A total of 102 and 11 bacterial isolates were obtained as epiphytic and endophytic bacteria, respectively, based on their distinct morphological characteristics. Subsequently, the epiphytic and endophytic bacteria were phylogenetically identified. The epiphytic bacteria belonged to six orders: *Actinomycetales, Alteromonadales, Bacillales, Enterobacteriales, Pseudomonadales*, and*Vibrionales*. Interestingly, the epiphytic bacteria that belonged to *Vibrionales* were commonly isolated from all of the macroalgal samples irrespective of the location and the season in which they were collected. Bacteria belonging to *Bacillales* were present only in the macroalgal samples that were collected during the pre-monsoon and monsoon seasons. Bacterial isolates belonging to *Pseudomonadales* and *Alteromonadales* were only isolated from *G*.*dura* collected from the Veraval coast while *Actinomycetales* and *Enterobacteriales* were only collected from *G. corticata* that was obtained from the Okha coast.

The endophytic bacteria are: *Allomonas enterica* (JQ665324), *Vibrio parahaemolyticus* (JQ665335), *Shewanella algae* (JN996469), *Pseudomonas aeruginosa* (JQ665348), *P. stutzeri* (JQ665358), *Micrococcus luteus* (JQ665283), *Bacillus cereus* (JQ665291), *B. licheniformis* (JQ665350), *V. sinaloensis* (JQ665310), *V. nigripulchritudo* (JQ665360), and *V. rotiferianus* (JQ665367). Among all of the endophytic bacteria, 10 bacterial isolates were isolated from the genus *Gracilaria* while *B. cereus* (JQ665291) was obtained from *U. fasciata*. *V. parahaemolyticus* was always found to be associated with *G. corticata*, whereas *S. algae* and *P. aeruginosa* were associated with *G. dura*, thereby showing evidence of algal host specificity.

### Identification of the AHL signals

In the MS/MS analysis, the activated natural compound [M + H]^+^ ion derived from the AHLs decomposed into specific ion products, including the [M + H- C_4_H_7_NO_2_ or M + H -101]^+^ that resulted from the neutral loss of homoserine lactone and an ion at m/z 102 corresponding to the protonated lactone (Decho et al., 2009). In the present study, seven different Gram-negative bacteria were found to produce different types of AHLs. The *S. algae* (JN996469) was found to produce several types of AHLs (C_4_-HSL, HC_4_-HSL, C_6_-HSL, 3-*oxo*-C_6_-HSL, and 3-*oxo*-C_12_-HSL), as shown in the Supplementary Datasheet, Figures S1A-D,H, (Table 1). *Photobacterium lutimaris* (JQ613504) was found to produce three types of AHLs (C_4_-HSL, HC_4_-HSL, C_6_-HSL) and each of the remaining bacterial isolates produced two types of AHLs, as shown in Table 1 and the Supplementary Datasheet 1, Figure 1. This experiment was repeated three times and the data were found to be reproducible.

**Table 1:** **Liquid chromatography–mass spectrometry/MS analysis of Gram-negative bacterial extracts for detecting *N*-acyl-homoserine lactone (AHL)**.

**AHLs**	**Parent ion**	**Fragmentation ions**		**Occurrence of AHLs in replicates**	**Bacterial strains**						
	**[M+H]+m/z**	**[M+H-chain]+m/z**	**[M+H-lactone ring]+m/z**		***Shewanella algae* (JN996469)**	***P. aeruginosa* (JQ613503)**	***Photobacterium* sp. (JQ613506)**	***P. lutimaris* (JQ613504)**	***Vibrio gallicus* (JQ665345)**	***V. fluvialis* (JQ665321)**	***V. parahaemolyticus* (JQ408396)**
C4-HSL	172.21	102.16	71.08	^***^	+	+	+	+	+	+	+
HC_4_-HSL	173.12	102.01	72	^***^	+	−	−	+	−	−	−
C_6_-HSL	200	102.01	99.04	^***^	+	−	+	+	−	−	−
3-*oxo*-C_6_-HSL	214	102.05	113.06	^**^	+	−	−	−	−	−	−
C_7_-HSL	213.98	101.93	113.05	^**^	−	−	−	−	−	+	−
C_8_-HSL	228.08	102.05	127.08	^**^	−	−	−	−	+	−	−
C10-HSL	256.28	102.16	155.37	^***^	−	−	−	−	−	−	+
3-*oxo* C12-HSL	298.17	102.16	197.44	^**^	+	+	−	−	−	−	−

*Using collision-induced dissociation (CID), the [M+H]^+^ ion, derived from the parent AHL molecule, decomposes into two “fragmentation ion” products corresponding to the lactone moiety (m/z 102) and the acyl-chain moiety [M+H–101]^+^ (Decho et al., 2009). m/z = mass/charge ratio. (+) indicates the detection of AHL and (−) absences of AHL. Except Shewanella algae, all the bacteria were isolated as epiphytic origin*.

### Effect of different AHLs on carpospore liberation from *G. dura*

**Figure 5:**
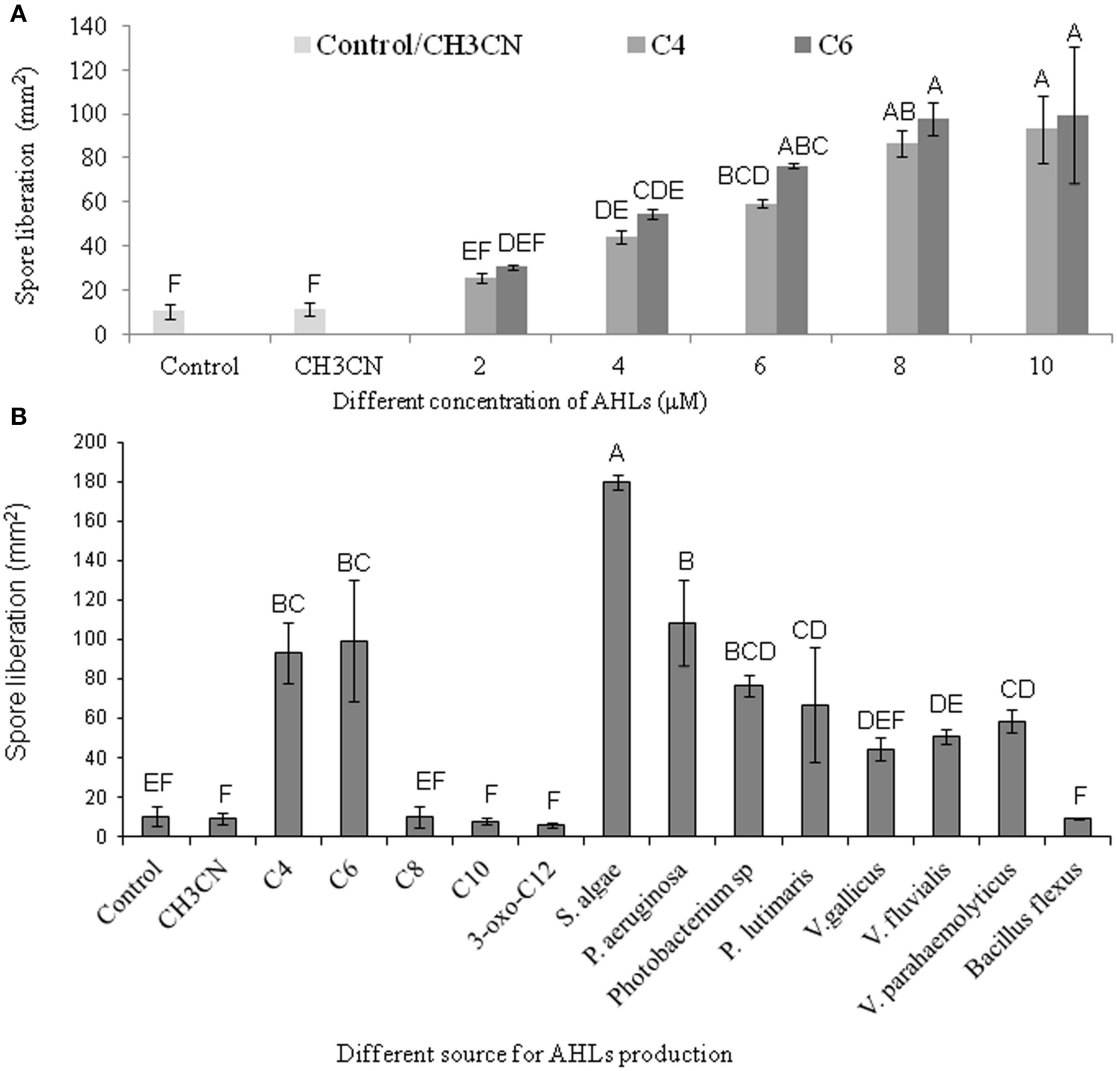
**Effect of different standard AHLs and Gram-negative bacterial isolates on carpospores liberation from *Gracilaria dura***. **(A)** Effect of different concentrations (2, 4, 6, 8, and 10 μM) of C_4_- and C_6_-HSL on carpospores liberation. **(B)** Effect of different AHLs standard at 10 μM, culture filtrates of Gram-negative bacterial isolates and *Bacillus flexus* on carpospores liberation. Bars indicate minima and maxima of three replicates. One Way ANOVA and Dunnett's *post-hoc* analysis showed significant differences at *p* > 0.01 for the C_4_-HSL, the C_6_-HSL, and culture filtrates of AHLs producing bacterial isolates. Effect of C_4_-HSL, C_6_-HSL, and culture filtrates of AHLs producing bacterial isolates (except *V. gallicus*) were significant at *p* < 0.001 whereas others had no effect (*P* > 0.05) in Bonferroni correction. Letter designation format was carried out with Tukey's HSD using JMP software, which means sharing the same letters were not different at *p* < 0.05. AHLs were dissolved in 100% CH_3_CN and working concentration of control was fixed at 0.04%.

AHL containing culture filtrates of seven Gram-negative bacteria and the AHL standards of C_4_- and C_6_-HSL were found to induce the liberation of carpospores in *G. dura* as compared to the control and the C_10_-, 3-oxo-C_12_-HSL, and culture filtrates of *B. flexus*. There was a positive correlation between different concentrations (2, 4, 6, 8, and 10 μM) of the C_4_- and C_6_-HSL and carpospore liberation from the cystocarps (Figure 5A). The culture filtrates of *S. algae* showed the ability to enhance carpospore liberation up to 179.625 ± 3.6 mm^2^ carpospores as compared to *P. aeruginosa*, which produced 108.375 ± 21.62 mm^2^ carpospores. The carpospores that were liberated with culture filtrates of *Photobacterium* sp., *P. lutimaris, V. gallicus, V*. *fluvialis*, and *V*. *parahaemolyticus* were 76.66 ± 5.07 mm^2^, 66.87 ± 28.97 mm^2^, 44.26 ± 6.06 mm^2^, 50.58 ± 3.74 mm^2^, and 62.83 ± 6.34 mm^2^, respectively. On the other hand, the standard C_4_- and C_6_- HSL yielded 93.333 ± 15.33 mm^2^ and 99.448 ± 30.94 mm^2^ carpospores, respectively (Figure 5B). One Way ANOVA and Dunnett's *post-hoc* analysis showed significant differences at *p* > 0.01 for the AHL standards and the bacterial culture filtrates. Additionally, Bonferroni correction was used to determine effect of AHLs and bacterial culture filtrates on carpospores liberation. Effect of C_4_-HSL, C_6_-HSL and culture filtrates of AHLs producing bacterial isolates (except *V. gallicus*) were significant at *p* < 0.001 whereas others had no effect (*P* > 0.05) in Bonferroni correction.

### Electrophoresis of protein profile of the AHL-treated cystocarps and the cystocarp-bearing plantlets

**Figure 6:**
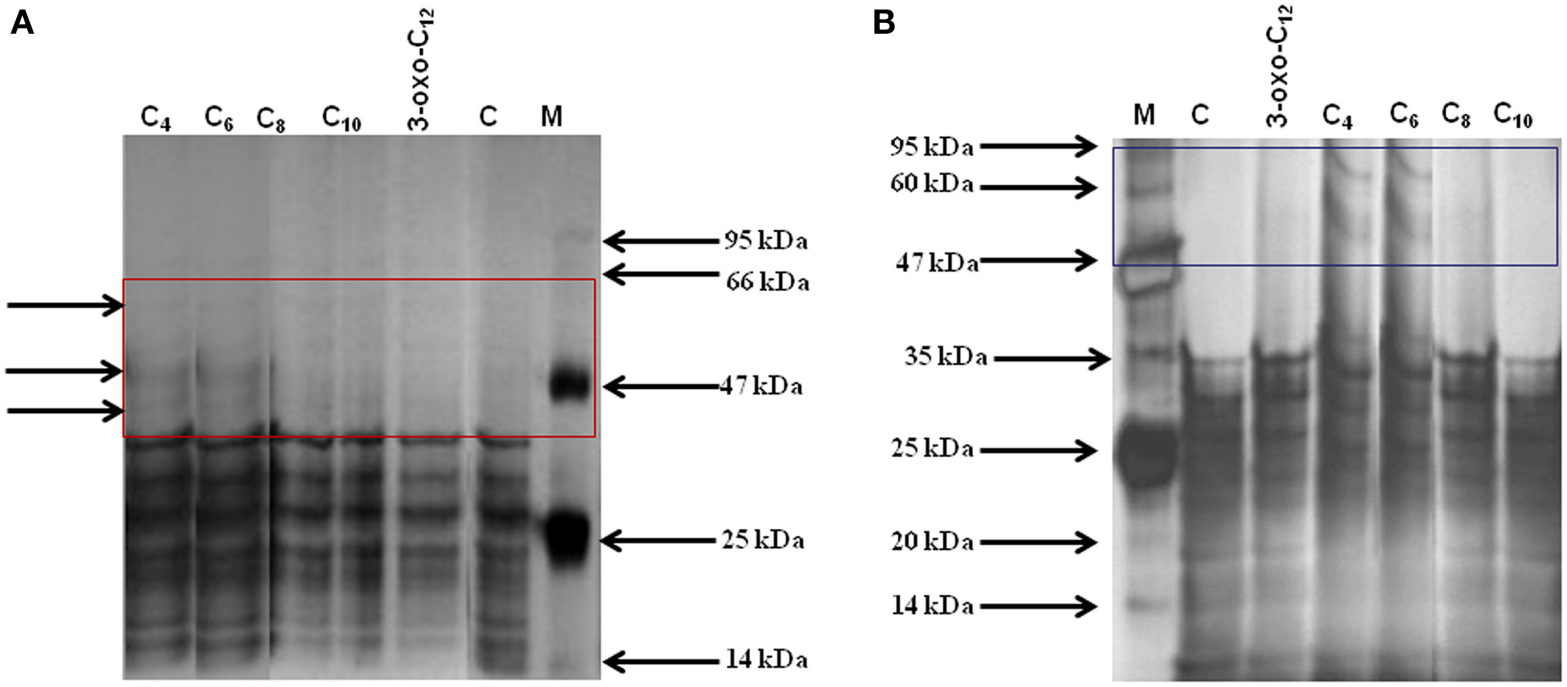
**Total protein profiling of AHLs treated cystocarps and cystocarp bearing plantlets of *G. dura with polyacrylamide gel electrophoresis***. **(A)** SDS-PAGE analysis of AHLs treated cystocarpic plantlets. **(B)** SDS-PAGE analysis of AHLs treated cystocarps. Different types of AHLs (C_4_, C_6_,C_8_, C_10_, and 3-oxo-C_12_-HSL) were used for experiments at concentration of 10 μM.

To understand the effect of different AHLs on carpospore liberation from the cystocarps of *G. dura*, the total protein profile of the AHL-treated cystocarps and the cystocarp-bearing plantlets were analyzed with polyacrylamide gel electrophoresis. Among all of the AHL-treated cystocarpic plantlets, those treated with C_4_- and C_6_-HSL showed three specific peptide bands with an approximate molecular weight of 45, 50, and 60 kDa, respectively (Figure 6A). In another experiment, the C_4_- and C_6_-HSL-treated cystocarps showed two specific peptide bands having an approximately molecular weight of 50 kDa and 60 kDa, respectively (Figure 6B). The C_8_-, C_10_-, and 3-oxo-C_12_-HSL-treated cystocarpic plantlets and the cystocarps and the control did not induce these specific protein bands. The specificity of the peptide bands was determined using Native-PAGE and it was found that these peptide bands represented three different proteins.

## Discussion

To obtain insight about the important role that seaweed-associated bacteria play in the host's life cycle, several types of epiphytic and endophytic bacteria were isolated from the *Ulva* and *Gracilaria* species. Subsequently, the isolated bacteria were screened for AHL production and their ability to liberate carpospores from the cyctocarp of *G. dura* was evaluated. The bacterial communities identified in this study were more or less similar to the bacterial communities identified from different seaweeds (Burke et al., 2011; Lachnit et al., 2011). Dominant bacterial members of *Gammaproteobacteria* were consistently encountered in all of the samples, seasons and locations thereby indicating their abundance in the marine environment. Similarly, Patel et al. (2003) and Tait et al. (2005) also reported *Gammaproteobacteria* as the dominant epiphytic bacteria associated with green macroalgae *Enteromorpha* and *Ulva* in samples taken from Wembury Beach, Devon, UK. The red macroalga *Amphiroa anceps* was also found to be a habitat for *Gammaproteobacteria* while *Bacteroidetes* and *Gammaproteobacteria* were found to be associated with another red alga *Corallina officinalis* (Huggett et al., 2006). The high abundance of *Gammaproteobacteria* on the surface of the seaweeds could be attributed to its tendency to form biofilms (Tait et al., 2009). Venter et al. (2004) and Giovannoni and Stingl (2005) analyzed planktonic communities found in seawater and they observed that *Gammaproteobacteria, Actinobacteria, Planctomycetes*, and *Bacillales* are commonplace in oceanic waters. Thus, phylogenetic studies of these epiphytic bacteria reveal that the recruitment of different bacterial communities that coexist with different seaweeds is of oceanic origin. A few previous reports have dealt with endophytic bacteria isolated from different macroalgae. In earlier studies, endophytic bacteria were isolated mainly for the chemical interactions from *Caulerpa, Codium, Bryopsis*, and *Penicillus* and those studies did not characterize their phylogenetic relevance (Please see the review of Goecke et al., 2010). Recently, Hollants et al. (2011) isolated endophytic bacteria belonging to *Flavobacteriaceae, Bacteroidetes*, and *Phyllobacteriaceae* from the siphonous green alga *Bryopsis hypnoides*, as well as*, Xanthomonadaceae, Gammaproteobacteria, Epsilonproteobacteria* and a new *Arcobacter* species isolated from *B. pennata*. Thus, limited information is available about the endophytic communities of seaweeds.

The age of the plantlets is also considered to be a significant inherent source of variation in seaweed-associated bacterial communities at spatial and temporal scales (Staufenberger et al., 2008; Goecke et al., 2010). It has been demonstrated that bacterial communities of young meristem and cauloid sections of different plantlets of the brown alga *Laminaria saccharina* were more similar to each other than the aging phyloid section of the same plantlets (Staufenberger et al., 2008). The present study has also confirmed the temporal variations of bacterial communities associated with macroalgal samples across seasons. We observed less seaweed-associated bacterial communities during the post-monsoon season as compared to the pre-monsoon and monsoon seasons (Figures 2, 3). During the pre-monsoon and monsoon seasons, the seaweed surfaces were also occupied by bacterial species of *Firmicutes*. Considering this fact, the present findings revealed that the bacterial species belonging to *Firmicutes* are highly variable while the bacterial species belonging to *Gammaproteobacteria* were found to be seaweed-philic but temporally variable. Despite these levels of variability, the epi- and endophytic communities are included in a sub-population of bacteria that were consistently associated with the *Ulva* and *Gracilaria* species. Such an observation provided evidence of core bacterial communities that have an important function in host macroalgae and will enhance our understanding of bacterial-host interactions in plant science.

In this study, *S. algae* was found to produce several types of AHLs (C_4_-, HC_4_-, C_6_-, 3-*oxo*-C_6_- and 3-*oxo*-C_12_-HSL); thus, its culture filtrates promoted carpospore liberation from *G. dura* as compared to the culture filtrates of *P. aeruginosa, Vibrio* and *Photobacteria* and the control. Weinberger et al. (2007) reported that C_4_-HSL potentially influenced the carpospore liberation capacity in *Acrochaetium* sp. While, the present study found that both C_4_- and C_6_-HSL equally contributed to carpospore liberation from *G. dura*. The positive correlation between different concentrations of C_4_- and C_6_-HSL and carpospore liberation from *G. dura* revealed that relative increases in the concentration of C_4_- and C_6_-HSL up to 10 μM also enhances carpospore liberation. The C_8_-, C_10_-, 3-oxo-C_12_-HSL and culture filtrates of Gram-positive bacterium *B*. *flexus* did not influence carpospore liberation thereby indicating selective regulation by C_4_- and C_6_-HSL (Figure 5). SDS-PAGE analysis of the total protein profile of the cystocarps of *G. dura* treated with C_4_- and C_6_-HSL revealed induction of specific peptide bands with an approximate molecular weight of 50 kDa and 60 kDa, whereas the cystocarpic plantlets treated with C_4_- and C_6_-HSL revealed three specific peptide bands with the approximate molecular weight of 45, 50, and 60 kDa as compared to the C_8_-, C_10_- and 3-oxo-C_12_-HSL-treated samples. Although, the AHLs identified from seven different bacteria in the present study are not quantified, a recent study reported 0.1–30 μM of AHLs are produced by Gram-negative biofilm-forming bacteria (Ahlgren et al., 2011). The effect of bacterial culture filtrates on the liberation of carpospores could not be limited to a particular species or even a specific genus level as different bacterial isolates of different orders showed dissimilar effects (Figure 5). The findings of the present study also suggested that the diffusion ability, stability and availability of AHLs around the cystocarpic plantlets are important factors for carpospore liberation in a natural environment and cross-talk between the seaweed-bacteria association. Tait et al. (2005) reported that short acyl chain molecules (C_6_-HSL and 3-hydroxy-C_6_-HSL) were diffused more quickly from agarose gel than longer acyl chain molecules, such as 3-*oxo*-C_10_-HSL. A similar finding was also observed in the higher plants. It has been reported that short side-chain AHL compounds are easily soluble in water and are actively taken up into plant roots as well as transported through shoots, as compared to long acyl side chain AHLs in barley and *A. thaliana* (Götz et al., 2007; von Rad et al., 2008; Hartmann et al., 2014; Sieper et al., 2014).

Macroalgal surfaces are living hosts and they perform an essential role in coastal ecosystems (Burke et al., 2011). *Firmicutes* have been found to be the second most abundant bacteria on these algal surfaces and they contribute to approximately 15–30% of dimethylsulfoniopropionate assimilation (Malmstrom et al., 2004). The high variability of bacterial communities associated with different species of *Ulva* and *Gracilaria*, or even among the same species, suggests that functional redundancy exists within these communities. This conclusion follows the redundancy hypothesis, which presumes that more than one species is capable of performing a specific role within an ecosystem (Naeem, 1998; Burke et al., 2011).

In conclusion, this study identified and characterized several epi- and endophytic bacterial communities associated with different taxa of *Ulva* and *Gracilaria*. It also demonstrated that some Gram-negative epi- and endophytic seaweed-associated bacteria produce different types of AHLs. The C_4_- and C_6_-HSL as well as the culture filtrate of seven AHL- producing Gram-negative bacteria were found to enhance carpospore liberation from the cyctocarps of *G. dura*. Thus, these bacterial isolates can effectively be used for mass carpospore liberation, even though the underpinning molecular mechanisms of this phenomenon are not well-understood yet. Additional biochemical and molecular studies are required to characterize their signaling mechanisms and those studies will serve to illuminate new avenues for further optimization of this phenomenon. Therefore, the evaluation of this molecule signaling cascade is our long-term goal and will be reflected in future publications.

## Author contributions

RPS, CRKR and BJ conceived and designed the work. RPS and RSB collected the samples and performed the experiments. CRKR and BJ analyzed the QS data. RPS and RSB conducted electrophoresis analysis and identified the bacteria associated with the seaweeds. RPS, CRKR and BJ wrote the manuscript. All of the authors contributed to the discussion and approved the final manuscript.

### Conflict of interest statement

The authors declare that the research was conducted in the absence of any commercial or financial relationships that could be construed as a potential conflict of interest.
