# Supplementary material for: Effect of quorum sensing signals produced by seaweed-associated bacteria on carpospore liberation from Gracilaria dura
Source: Front Plant Sci. 2015 Mar 4;6:117. doi: 10.3389/fpls.2015.00117 (PMC4349058; doi:10.3389/fpls.2015.00117)
Supplement: Supplementary file 1 [file DataSheet1.PDF]

Figure S1 LC-MS/MS-CID spectra of AHLs analyzed from different culture filtrates of Gram-negative bacterial strains. (a) spectra for HC_4-HSL, (b) spectra for C_4-HSL, (c) spectra for C_6-HSL, (d) spectra for 3-oxo-C_6-HSL, (e) MS /MS spectra for C_7-HSL, (f) MS /MS spectra for C_8-HSL, (g) MS /MS spectra for C_{10}-HSL and (h) MS /MS spectra for 3-oxo-C_{12}-HSL. *m/z* = mass/charge ratio. Y axis is "Relative intensities"

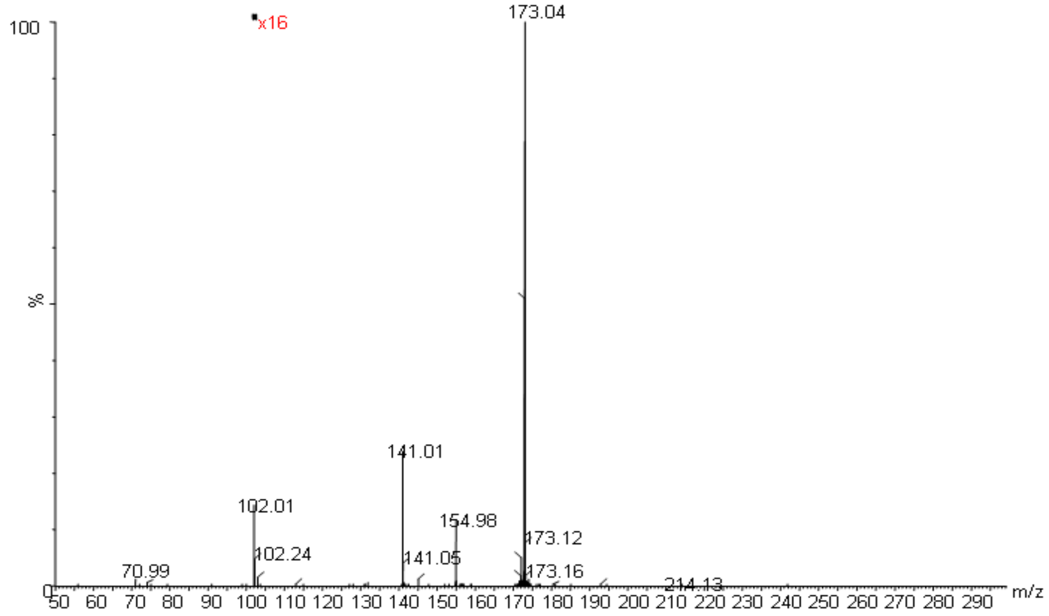

Fig. S2A

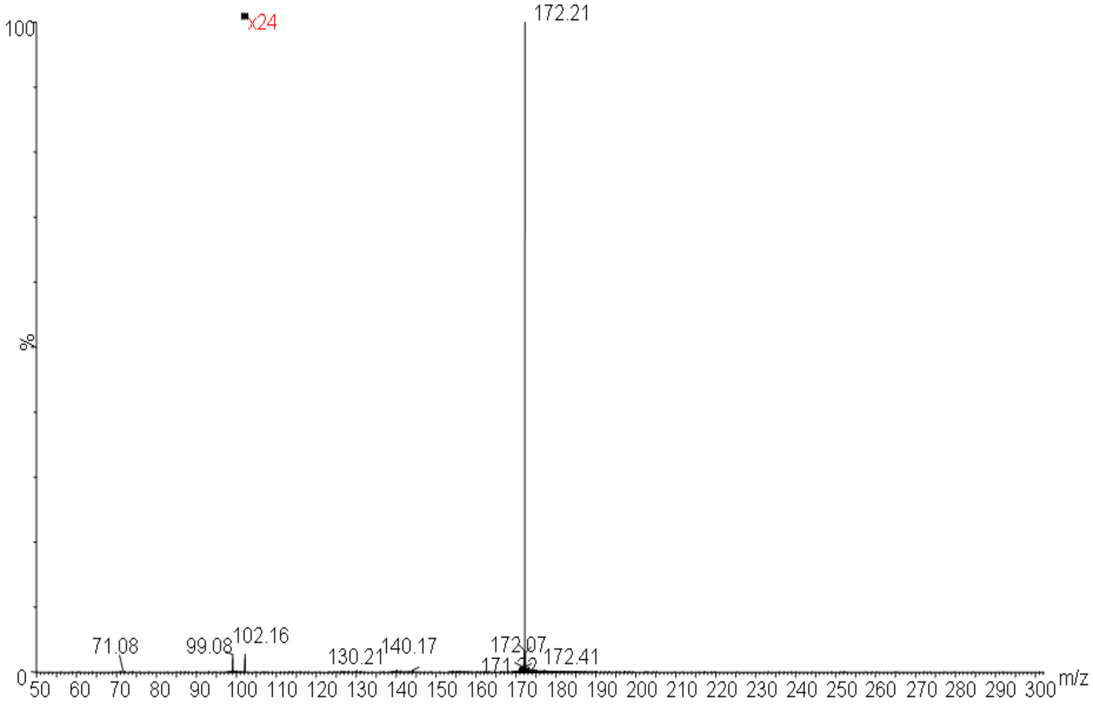

Fig. S2B

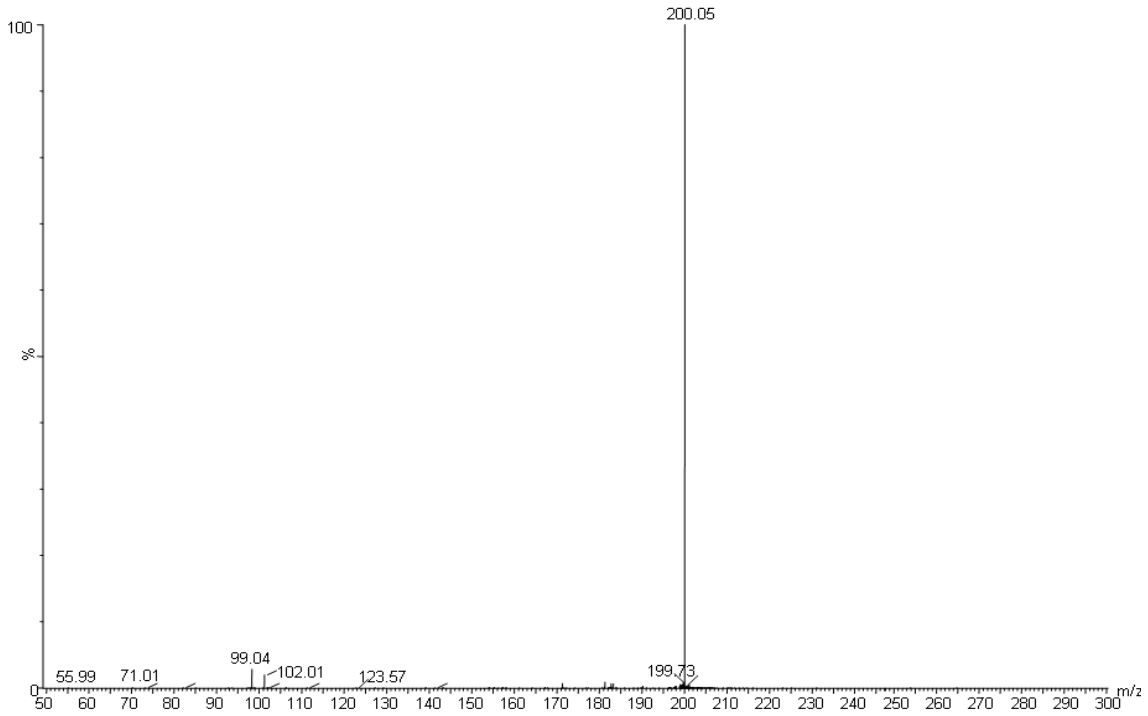

Fig. S2C

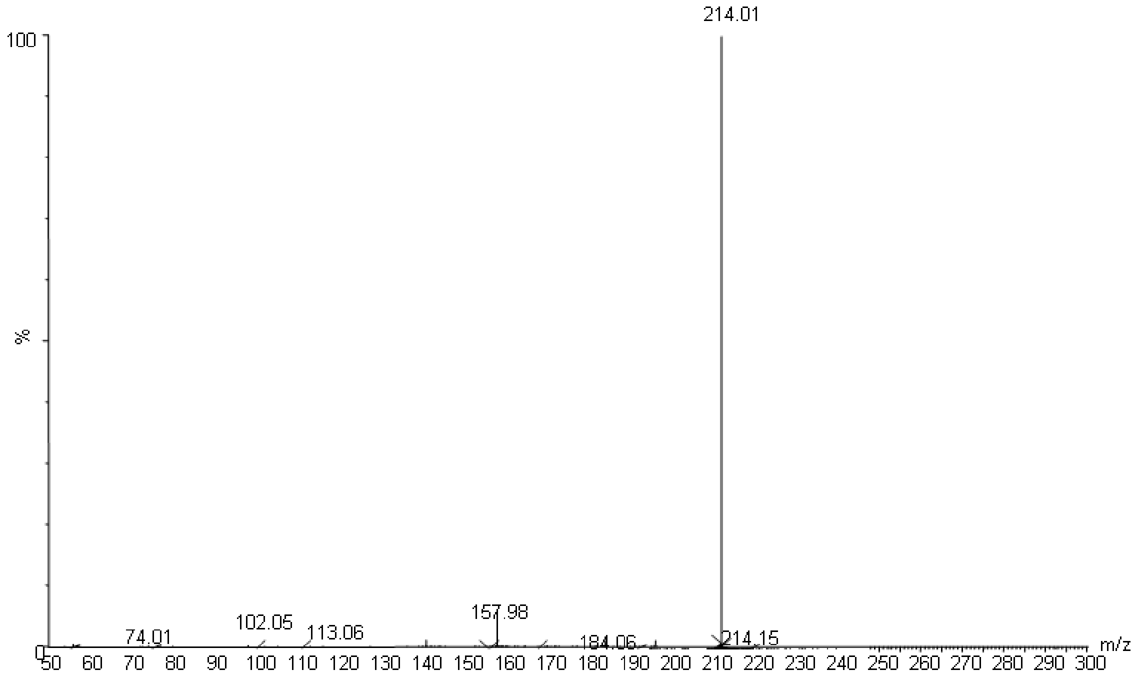

Fig. S2D

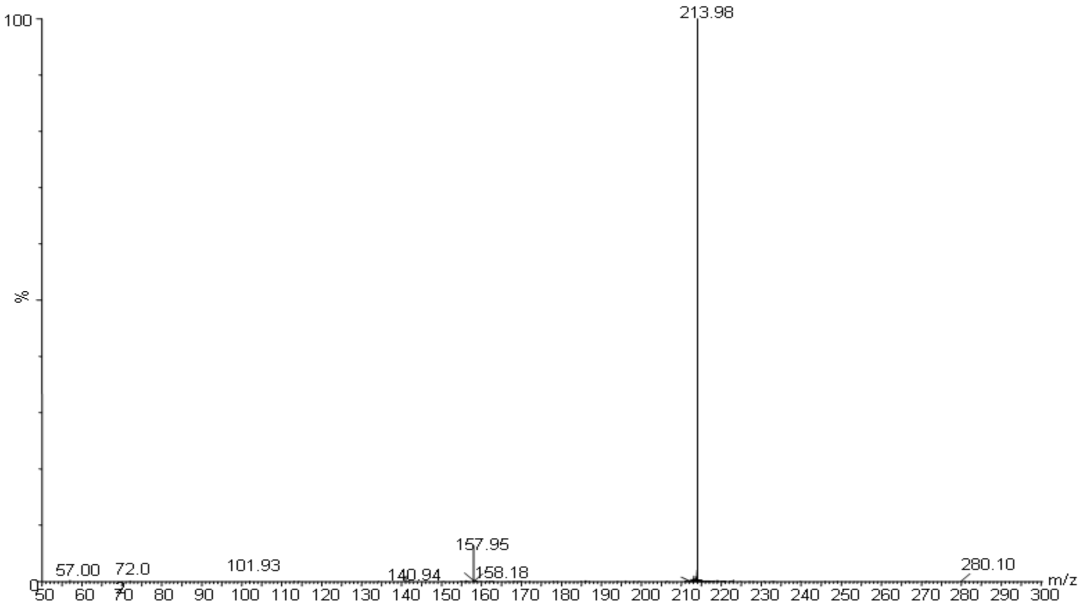

Fig. S2E

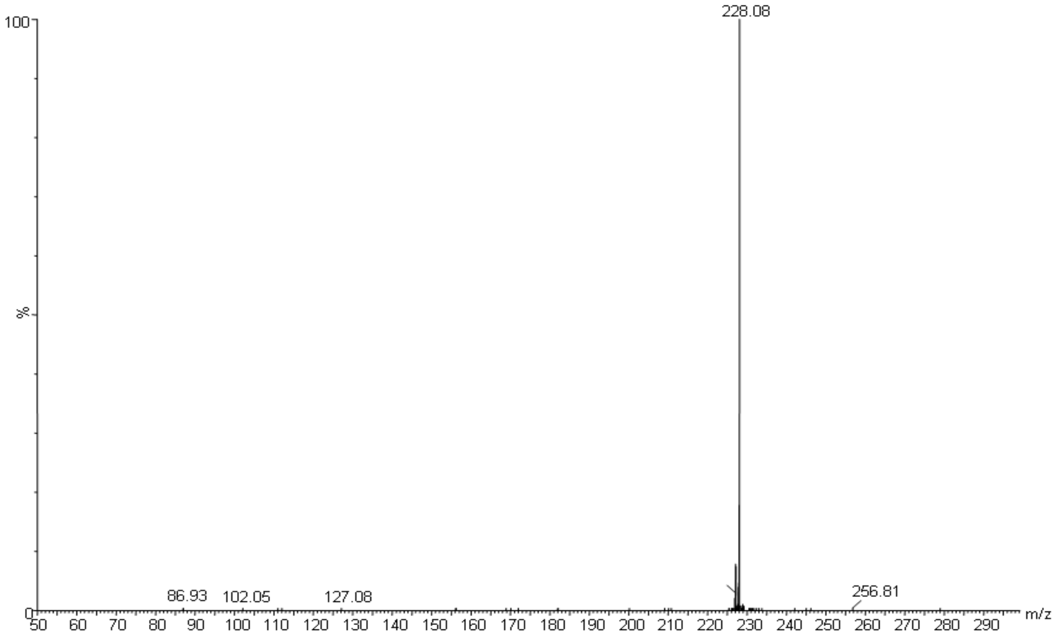

Fig. S2F

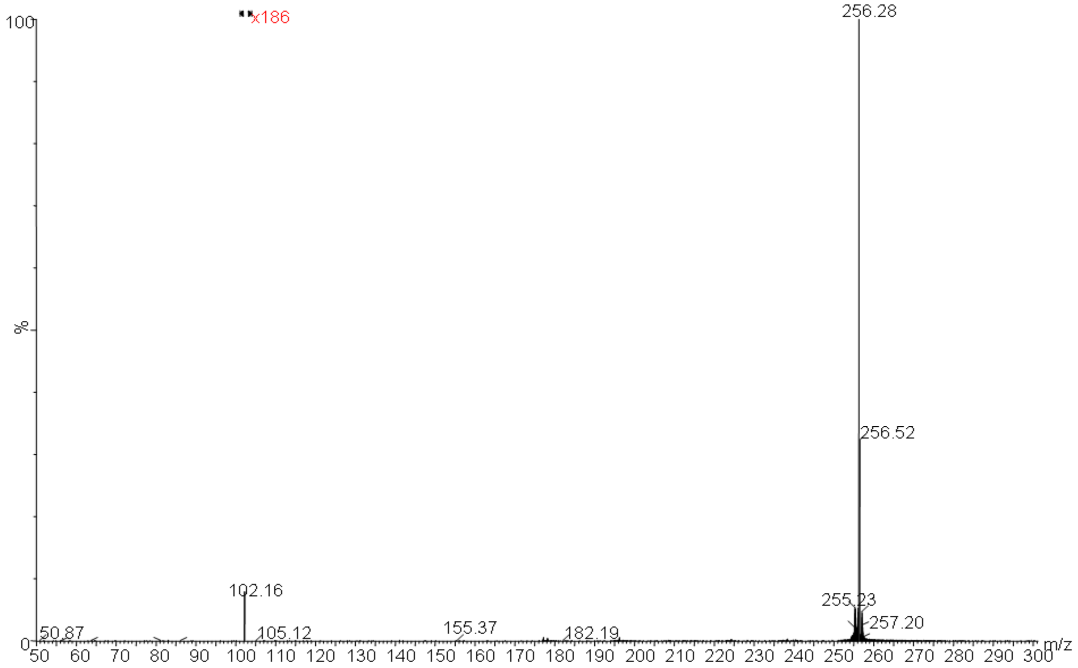

Fig. S2G

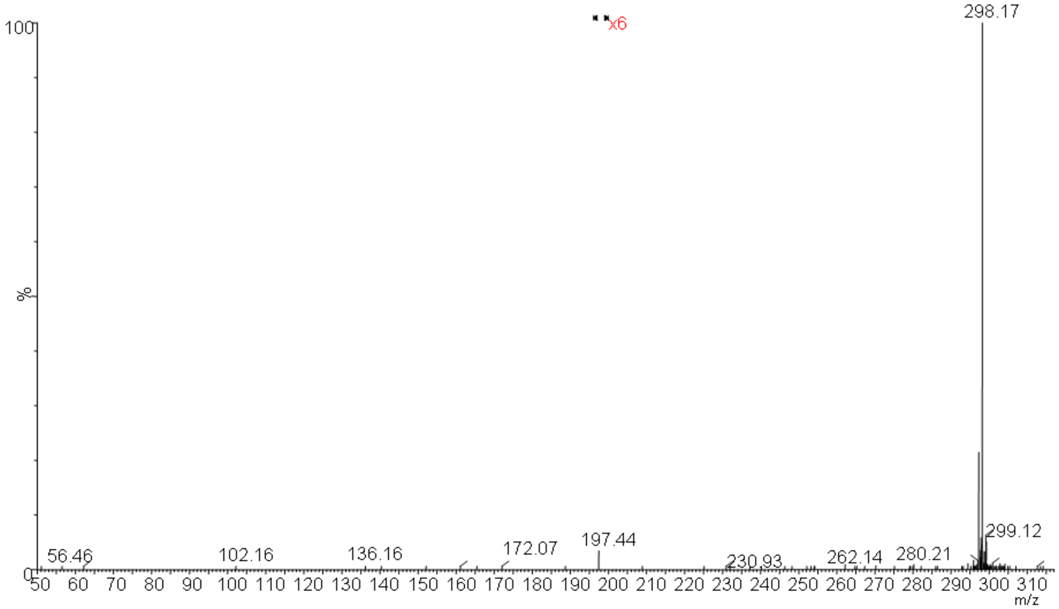

Fig. S2H
