# Supplementary material for: Effect of quorum sensing signals produced by seaweed-associated bacteria on carpospore liberation from Gracilaria dura
Source: Front Plant Sci. 2015 Mar 4;6:117. doi: 10.3389/fpls.2015.00117 (PMC4349058; doi:10.3389/fpls.2015.00117)
Supplement: Supplementary file 2 [file Table1.PDF]

Supplementary Table 1. Physio-chemical parameter. Different parameters including average pH, temperature and salinity were measured during collection time.

Prameter	Pre-monsoon		Monsoon		Post-monsoon	
	Veraval	Okha	Veraval	Okha	Veraval	Okha
pH	8.2	8.2	8.1	8.2	7.6	8.1
Temperature (ºC)	29	32	29	31	24	28
Salinity (‰)	36	39	22	28	36	35
