# Supplementary material for: Effect of quorum sensing signals produced by seaweed-associated bacteria on carpospore liberation from Gracilaria dura
Source: Front Plant Sci. 2015 Mar 4;6:117. doi: 10.3389/fpls.2015.00117 (PMC4349058; doi:10.3389/fpls.2015.00117)
Supplement: Supplementary file 3 [file Table2.PDF]

Supplementary Table 2. Different combinations of solvents were used for the separation of different AHLs

Time	A (Water + 0.1% acetic acid, v/v)	B (Methanol + 0.1% acetic acid, v/v)	Flow rate (µl)
0	95	5	0.30
5	95	5	0.30
30	5	95	0.30
40	5	95	0.30
41	95	5	0.30
60	95	5	0.30
