# Supplementary material for: Effect of quorum sensing signals produced by seaweed-associated bacteria on carpospore liberation from Gracilaria dura
Source: Front Plant Sci. 2015 Mar 4;6:117. doi: 10.3389/fpls.2015.00117 (PMC4349058; doi:10.3389/fpls.2015.00117)
Supplement: Supplementary file 4 [file Table3.PDF]

Table S3. Summary of bacterial types were observed from different microbiological media (Zobell marine agar 2216, Simmons citrate, thiosulfate citrate bile salts sucrose (TCBS), xylose, lysine, deoxycholate (XLD) agar and pseudomonas agar)**.**

		Pre- monsoon, number of bacteria isolated from each frond			Total bacteria isolated (morphologically distinct)	Monsoon, number of bacteria isolated from each frond			Total bacteria isolated (morphologically distinct)	Post- monsoon, number of bacteria isolated from each frond			Total bacteria isolated (morphologically distinct)
Location	Species	Frond-1	Frond-2	Frond-3		Frond-1	Frond-2	Frond-3		Frond-1	Frond-2	Frond-3	
Veraval	*U. fasciata*	5	6 (En,1)	3 (En,1)	6 (En,1)	6	7	8	8	5	5	4	5
	U. lactuca	4	7	4	7	7	5	0	7	4	4	5	5
	G. dura	2	6 (En,1)	5 (En,1)	6 (En,1)	4 (En,1)	7 (En,1)	6	7 (En,2)	0	3	4	4
	G. corticata	4 (En,1)	2	1	4 (En,1)	10 (En,1)	9 (En,1)	5 (En,1)	10 (En,2)	6 (En,1)	9 (En,1)	8 (En,1)	9 (En,1)
Okha	*U. fasciata*	3	5	7	7	6	5	6	6	5	4	5	5
	G. corticata	4 (En,1)	6 (En,1)	5 (En,1)	6 (En,1)	6	6 (En,1)	6	6 (En,1)	5	5 (En,1)	4 (En,1)	5 (En,1)

Only morphologically distinct bacteria were used in the study and were identified by 16S rDNA analysis. Total 11 endophytic bacterial were isolated. Some endophytic bacteria were always isolated from same type of algal frond. En, is represented for endophytic. Three different fronds were collected from each location and a number of bacteria were isolated from each frond. Finally, total bacteria isolated, represented total number of bacteria which were morphological distinct collected from three different fronds of same species.
